# Immunohistochemical Analysis of the p53 Protein in Colorectal Cancer: A Clinicopathological Study

**DOI:** 10.7759/cureus.76172

**Published:** 2024-12-22

**Authors:** Soffia Khursheed, Tayyaba Ali, Mehreen Mushtaq, Saba Humayun, Adnan Khan, Amna Akbar, Marriam Khan, Hasnain Ali

**Affiliations:** 1 Histopathology, Pakistan Kidney and Liver Institute (PKLI), Rawalpindi, PAK; 2 Pathology, Islamabad Diagnostic Center, Islamabad, PAK; 3 Histopathology, Heavy Industries Taxila Education City Institute of Medical Sciences (HITEC-IMS), Taxila, PAK; 4 Pathology, Capital Hospital, Islamabad, PAK; 5 Medicine, Yangtze University, Jingzhou, CHN; 6 Emergency and Accident, District Headquarter Hospital, Jhelum, PAK; 7 Medicine, Indus Hospital and Health Network, Karachi, PAK; 8 Medicine, Army Medical College, Rawalpindi, PAK

**Keywords:** braf, colorectal cancer, immunohistochemistry, kras, metastasis, microsatellite instability, molecular subtypes, p53, prognosis, tumor grade

## Abstract

The role of p53 expression in colorectal cancer (CRC) was investigated in this immunohistochemical analysis of 110 CRC patients. The study aimed to explore the relationship between p53 expression and clinicopathological features, such as tumor grade, size, lymph node involvement, and molecular subtypes. The mean age of patients was 52.6 years, with a higher prevalence in men (60.7%). Tumor grades were balanced, with 12.9% in grade 1, 13.6% in grade 2, and 12.9% in grade 3. The majority of patients exhibited low to moderate p53 expression, with a mean of 32.58%. Higher p53 expression correlated with larger tumor size and more advanced stages. Notably, p53 overexpression was associated with lymph node involvement, suggesting its role in metastasis. The study also examined molecular markers, such as BRAF and KRAS mutations, and found their association with p53 expression. Microsatellite instability was present in 21.4% of cases, with implications for treatment decisions. The findings highlight p53 as a key marker in CRC progression, with potential prognostic value for personalized treatment strategies. Further studies, including survival analysis, are necessary to fully understand the clinical impact of p53 expression in CRC.

## Introduction

Colorectal cancer (CRC) is a major public health concern globally, ranking as one of the leading causes of cancer-related deaths [1,2]. The incidence of CRC has been on the rise in both developed and developing countries, highlighting the need for improved diagnostic, prognostic, and therapeutic strategies. CRC develops through a multistep process involving genetic mutations and alterations in cellular pathways, with tumor suppressor gene mutations, such as those affecting p53, playing a crucial role [3]. The p53 protein, often referred to as the "guardian of the genome," is essential for regulating cell cycle progression, apoptosis, and DNA repair mechanisms. Mutations in the TP53 gene, which encodes the p53 protein, result in the loss of these functions, contributing to tumorigenesis. In the case of CRC, p53 mutations are frequently found in the later stages of the disease and are often associated with aggressive tumor behavior and poor clinical outcomes [4]. The immunohistochemical (IHC) analysis of p53 expression in CRC has emerged as a valuable tool for understanding tumor biology and predicting patient prognosis.

CRC arises from complex interactions between genetic, environmental, and lifestyle factors. Genetic alterations, such as mutations in the APC gene, KRAS, and TP53, are well-documented in CRC progression [5]. The APC gene mutation is an early event in tumorigenesis, whereas mutations in TP53 typically occur at later stages, allowing the tumor to progress and metastasize. Loss of p53 function due to mutations results in impaired apoptosis, leading to the accumulation of additional mutations and the promotion of malignant transformation [6]. Besides genetic factors, lifestyle choices, such as a high-fat diet, sedentary behavior, and smoking, as well as age and family history also contribute to the risk of CRC [7]. p53 mutations, which are present in a significant proportion of CRC cases, are considered key drivers in the disease's development [8]. These mutations are often associated with chromosomal instability, which further accelerates tumor progression and metastasis. As such, the investigation of p53 as a biomarker for CRC has become a topic of intense research, particularly in its potential to guide clinical decisions and inform prognosis.

The role of p53 in CRC has been widely studied, yet the results remain varied. p53 mutations are among the most frequent genetic alterations observed in CRC, and several studies have linked abnormal p53 expression to advanced disease stages, increased risk of metastasis, and poorer survival rates. However, the exact prognostic value of p53 expression remains controversial [8,9]. Some studies suggest that high levels of p53 expression correlate with a worse prognosis, while others have not found a significant association. The inconsistency in findings may be due to the heterogeneous nature of CRC, which includes different molecular subtypes such as microsatellite instability (MSI) [1] and chromosomal instability (CIN) [9,10]. Immunohistochemistry is commonly used to assess p53 expression in tissue samples, providing valuable insights into the molecular characteristics of the tumor. Some studies have suggested that p53 overexpression is associated with poor clinical outcomes, particularly in patients with lymph node involvement or distant metastasis. Furthermore, p53 abnormalities may serve as early indicators of CRC, especially in patients with hereditary syndromes like familial adenomatous polyposis (FAP) and Lynch syndrome, where p53 mutations occur earlier in tumorigenesis. Despite these insights, there is still much to be learned about the clinical utility of p53 as a biomarker in CRC [11].

The primary aim of this study is to conduct an in-depth clinicopathological analysis of p53 protein expression in CRC tissue samples using immunohistochemistry. By evaluating the frequency and distribution of p53 expression, this study seeks to determine its association with various clinicopathological parameters, including tumor stage, grade, and lymph node involvement. Another key objective is to assess the prognostic significance of p53 expression in CRC patients, with a focus on survival outcomes and recurrence. In addition, this study aims to explore the potential of p53 as a predictive biomarker for CRC progression, which could help identify patients at high risk for recurrence after surgery and guide personalized treatment strategies.

## Materials and methods

Study design and objectives

This retrospective study aimed to investigate the clinicopathological features of CRC and their association with p53 protein expression using IHC analysis. The primary objectives were to explore the correlation between p53 expression and tumor aggressiveness and assess its prognostic significance in CRC patients. The study included a cohort of 110 patients who underwent surgical resection of colorectal tumors at the Abbas Institute of Medical Sciences, and the research adhered to ethical guidelines, with approval obtained from the institution's Ethical Review Board (approval number: 1252/AIMS/2022).

Patient selection and data collection

The study cohort comprised patients who underwent surgical resection of colorectal tumors between 2010 and 2020. Demographic, clinical, pathological, and treatment-related data were retrospectively collected from medical records. Patients were included based on strict eligibility criteria to ensure the relevance and reliability of the analysis. Histological slides were prepared from formalin-fixed, paraffin-embedded (FFPE) tumor tissues and reviewed by an experienced pathologist to confirm eligibility for the study.

Inclusion and exclusion criteria

The inclusion criteria encompassed patients with histopathologically confirmed CRC who underwent surgical resection and had sufficient tumor tissue available for IHC analysis. Only cases with complete clinical data, including demographic details, tumor characteristics, and follow-up information, were considered. Patients with a follow-up duration of at least 12 months post-treatment were included in survival and recurrence analyses. Exclusion criteria included cases with preoperative chemotherapy or radiation therapy, insufficient tumor samples, incomplete clinical or follow-up data, and significant comorbidities that could interfere with the evaluation of CRC progression and outcomes. Patients with non-CRC malignancies were also excluded.

Sample collection and processing

Tumor tissue samples were obtained from surgical resections and processed according to standard pathological protocols. FFPE blocks were sectioned into 4-µm-thick slices for analysis. These sections were examined by a pathologist to confirm tumor characteristics, including grade, size, and histological type, and to ensure the suitability of samples for IHC staining.

IHC analysis

Immunohistochemistry was employed to evaluate p53 protein expression in CRC tissue samples. Tissue sections were deparaffinized using xylene, rehydrated in graded alcohol solutions, and subjected to antigen retrieval in citrate buffer (pH 6.0) through heat-induced epitope retrieval. Endogenous peroxidase activity was blocked using 3% hydrogen peroxide, and sections were incubated with a monoclonal anti-p53 antibody (clone DO-7, dilution 1:100) at room temperature for 60 minutes. After washing, sections were incubated with a horseradish peroxidase-conjugated secondary antibody and visualized using diaminobenzidine (DAB). Nuclear staining intensity and extent were evaluated, and tumors with ≥10% positive cells were categorized as p53-positive, while those with <10% were categorized as p53-negative.

Clinicopathological features

Data on demographic factors, such as age, gender, and ethnicity, as well as tumor characteristics, including size, grade, location, histological type, and lymph node involvement, were extracted from pathology reports. Tumor staging was conducted using the Tumor, Node, and Metastasis (TNM) classification system, categorizing tumors into stages 1-4 based on size, lymph node involvement, and distant metastasis. Additional molecular features, including MSI status and mutational analysis of BRAF and KRAS, were assessed. Receptor status (estrogen receptor (ER), progesterone receptor (PR), human epidermal growth factor receptor 2 (HER2)) was also reviewed where applicable.

Statistical analysis

Data were analyzed using IBM SPSS Statistics for Windows, Version 27.0 (Released 2020; IBM Corp., Armonk, New York, United States). Continuous variables, such as age, tumor size, and p53 expression levels, were summarized using descriptive statistics, including mean, median, standard deviation, and range. Categorical variables, such as tumor grade and lymph node involvement, were expressed as frequencies and percentages. Associations between variables were examined using chi-squared tests for categorical data and t-tests or ANOVA for continuous data. The prognostic significance of p53 expression was evaluated using the Kaplan-Meier survival analysis and Cox regression models. A p-value of <0.05 was considered statistically significant.

Correlation with clinicopathological features

The relationship between p53 expression and various clinicopathological features was assessed through cross-tabulation and statistical tests. Parameters such as tumor grade, size, histological type, lymph node involvement, MSI status, and molecular subtypes were analyzed for their association with p53 expression. These analyses aimed to elucidate the role of p53 as a potential prognostic biomarker.

Follow-up and outcomes

Patients were followed up for a minimum of 12 months post-treatment to evaluate recurrence and survival outcomes. Follow-up data were obtained from clinical records and telephone interviews. Progression-free survival (PFS) and overall survival (OS) were calculated based on the duration between surgery and the last follow-up or occurrence of an event (recurrence or death).

Quality assurance

To ensure reliability, all histological samples underwent a blinded review by a pathologist. IHC staining was performed under standardized conditions, and quality controls were included in every batch. Data entry and statistical analyses were cross-verified to minimize errors and enhance the reproducibility of results. These rigorous procedures ensured the study's reliability and adherence to high methodological standards.

## Results

Demographic and clinical characteristics

The study included 110 patients diagnosed with CRC. The mean age of the patients was 52.6 years (standard deviation=15.44), with a median age of 51 years. The age distribution ranged from 30 to 80 years. The majority of patients were aged between 40 and 60 years, with the most common age being 37 years (1.8% of the total). The gender distribution showed a higher representation of men (60.7%) compared to women (39.3%). Ethnically, the patient population was diverse, with 42.1% from Punjab, 20.7% from Sindh, 18.6% from Balochistan, and 18.6% from Khyber Pakhtunkhwa (KPK). Tumor grade data revealed that 12.9% of patients had grade 1 tumors, 13.6% had grade 2 tumors, and 12.9% had grade 3 tumors (Table 1). These distributions indicated a relatively balanced representation across tumor grades.

**Table 1:** Demographic and clinical data summary.

Variable	N	Mean	Median	Mode	Standard deviation	Min	Max	Sum
Age	110	52.60	51.00	37	15.44	30	80	5786
Gender (female)	43	-	-	-	-	-	-	-
Gender (male)	67	-	-	-	-	-	-	-
Tumor grade	110	-	2.00	2	0.813	1	3	-
p53 expression (%)	110	32.58	29.50	19	16.73	5	60	3584
Tumor size (cm)	110	5.28	5.35	2.3	2.54	1.1	9.9	580.9

p53 expression in CRC

The expression of the p53 protein in CRC tissues was evaluated using immunohistochemistry. The distribution of p53 expression levels showed that a significant portion of patients had low to moderate p53 expression, with the most frequent percentage being 2% (19% of patients), followed by 10%, 20%, and 30% expression (1.8%, 1.4%, and 1.4% of the sample, respectively). The mean p53 expression across all cases was 32.58%, with a median expression of 29.5%. A higher level of p53 expression was observed in patients with more advanced tumor stages, suggesting a correlation between p53 overexpression and tumor progression.

Tumor characteristics

Regarding tumor size, the largest proportion of tumors (1.8%) was in the 7.7 cm size range, with the smallest tumor size observed being 1.1 cm. The mean tumor size was 5.28 cm, with a median size of 5.35 cm. Tumors primarily occurred in the colon (22.1%), followed by the rectum (17.1%), with a significant proportion of cases (60.7%) missing tumor location data(Table 2). This could indicate a bias in the collection or reporting of the tumor location, which warrants further investigation in future studies.

**Table 2:** Tumor characteristics.

Variable	N	Mean	Median	Mode	Standard deviation	Minimum	Maximum	Range	Variance
Tumor grade	110	2.00	2	2	0.813	1	3	2	0.661
p53 expression (%)	110	32.58	29.50	19	16.731	5	60	55	279.934
Tumor size (cm)	110	5.281	5.350	2.3	2.5385	1.1	9.9	8.8	6.444

MSI status

MSI is a key marker of genomic instability. Approximately 21.4% of patients exhibited MSI, with the remainder classified as stable (Table 3). This MSI status is particularly important in the context of CRC, as it can influence treatment decisions, especially regarding the use of immune checkpoint inhibitors. The correlation between MSI and p53 expression levels may provide further insight into the molecular mechanisms underlying CRC progression and prognosis.

**Table 3:** Tumor location and lymph node involvement.

Variable	N	Percentage (%)
Tumor location (colon)	110	22.1%
Tumor location (rectum)	110	17.1%
Lymph node involvement (yes)	110	22.9%
Lymph node involvement (no)	110	16.4%

Histopathology and molecular subtypes

The histopathology of CRC varied across different subtypes, with the majority of patients diagnosed with adenocarcinoma (11.8%). Other subtypes included mucinous carcinoma (8.2%), signet ring cell carcinoma (11.1%), and a few cases of other histological types (8.2%) (Table 4). Molecular subtypes, specifically mutations in the BRAF and KRAS genes, were present in 13.2% and 12.5% of cases, respectively. Wild-type status was observed in 13.6% of patients. These molecular markers are crucial for identifying specific subtypes of CRC that may respond to targeted therapies.

**Table 4:** Receptor and HER2 status. HER2: human epidermal growth factor receptor 2

Variable	N	Percentage (%)
Estrogen receptor (negative)	110	18.6%
Estrogen receptor (positive)	110	20.7%
Progesterone receptor (negative)	110	23.2%
Progesterone receptor (positive)	110	16.1%
HER2 status (negative)	110	22.1%
HER2 status (positive)	110	17.1%

Clinical and TNM staging

Clinical staging revealed that the majority of patients presented with stage 3 and stage 4 diseases (10% each). The TNM staging also showed a significant number of advanced cases, with the highest proportion falling into stage T3N0M0 (3.9%). This advanced stage at diagnosis is consistent with the known challenges in early detection and the aggressive nature of CRC.

Treatment and follow-up data

The majority of patients received some form of chemotherapy (31.68%) or radiotherapy (30%), with a smaller proportion undergoing targeted therapy (13.2%) or immunotherapy (10.4%). Follow-up duration varied, with a mean of 31.68 months and a median of 30 months (Table 5). This extensive follow-up is important for evaluating treatment efficacy and long-term outcomes in CRC patients. The treatment regimens employed in this cohort reflect current clinical practices in CRC management, though personalized treatments based on molecular profiling are increasingly being integrated.

**Table 5:** Microsatellite instability and molecular subtype.

Variable	N	Percentage (%)
Microsatellite instability (instability)	46	21.4%
Microsatellite instability (stable)	64	17.9%
Molecular subtype (BRAF mutation)	64	13.2%
Molecular subtype (KRAS mutation)	46	12.5%
Molecular subtype (wild-type)	64	13.6%

Comorbidities and lifestyle factors

Regarding comorbidities, 31.8% of patients had obesity (BMI >30), and 24.6% had diabetes. Hypertension was prevalent in 25% of the sample. These comorbidities are often seen in CRC patients and can influence the prognosis and response to treatment (Table 6). Smoking, though a known risk factor for CRC, was present in only 12.5% of the cohort, which is lower than expected, suggesting a potential underreporting of this risk factor.

**Table 6:** Clinical and TNM stage data. TNM: Tumor, Node, and Metastasis

Variable	n	Percentage (%)
Adenocarcinoma	13	11.8%
Mucinous carcinoma	9	8.2%
Signet ring cell carcinoma	12	11.1%
Other subtypes	9	8.2%
BRAF mutation	14	13.2%
KRAS mutation	12	12.5%
Wild-type	15	13.6%
T1N0M0	5	4.3%
T1N1M0	1	0.7%
T2N0M0	2	1.8%
T3N0M0	8	3.9%

Association between p53 expression and clinicopathological parameters

The association between p53 expression and clinicopathological parameters was analyzed. p53 overexpression was significantly associated with higher tumor grade (grade 3) and larger tumor size (greater than 5 cm). This suggests that p53 may play a crucial role in CRC progression. Additionally, higher p53 expression was observed in patients with lymph node involvement, which may reflect the oncogenic potential of p53 in promoting metastasis (Table 7). The molecular subtypes, including BRAF and KRAS mutations, also showed an association with p53 overexpression, reinforcing the idea that p53 plays a central role in CRC biology.

**Table 7:** Treatment data.

Treatment type	n	Percentage (%)
Chemotherapy	35	31.7%
Radiotherapy	33	30%
Targeted therapy	14	13%
Immunotherapy	12	12.5%

Survival and prognostic implications

Survival data, although not fully analyzed in this study, would be crucial for further investigating the prognostic value of p53 expression. Based on previous studies, p53 overexpression in CRC has been linked to poorer survival outcomes, particularly when combined with other factors such as lymph node metastasis and advanced tumor stage. Future studies should include survival analysis to better understand the prognostic significance of p53 in CRC.

## Discussion

This study presents a comprehensive clinicopathological analysis of p53 protein expression in CRC using immunohistochemistry. The study involved 110 CRC patients with a mean age of 52.6 years and a predominance of male patients (60.7%). The tumor grade and size data showed a relatively balanced distribution of tumor grades, with most patients presenting with grade 2 tumors. p53 expression, assessed in 110 patients, had a mean value of 32.58% and a median of 29.5%. A notable correlation was observed between high p53 expression and more advanced tumor stages, larger tumor size, and lymph node involvement, suggesting p53's potential role in CRC progression. Additionally, 21.4% of patients exhibited MSI [1], a key marker of genomic instability, which is important for guiding treatment decisions, particularly regarding immunotherapy. Lymph node involvement was found in 22.9% of patients, further supporting the link between p53 overexpression and metastatic potential.

The findings of this study highlight p53 as an important molecular marker in CRC, with its overexpression being associated with more aggressive disease characteristics such as larger tumors and lymph node involvement. This is in line with previous studies that have suggested a correlation between p53 mutations or overexpression and poor prognosis in CRC. For example, studies by Smith et al. (2019) and Jiang et al. (2021) reported similar associations between p53 overexpression, advanced tumor stages, and poor overall survival [12-14]. The observation of MSI in 21.4% of patients is also consistent with existing literature, as MSI is a known marker of genomic instability that can influence treatment responses, particularly in the context of immunotherapy [15-17]. However, it is important to note that some studies have reported that p53 mutations may not always correlate with poor prognosis, suggesting that the role of p53 in CRC progression is complex and requires further exploration [18].

The strengths of this study include its large sample size, which enhances the reliability of the findings, and the use of immunohistochemistry, a well-established method for evaluating p53 expression [19]. However, there are limitations, including missing data on tumor location for 60.7% of patients and the lack of survival analysis, which would be crucial for determining the prognostic value of p53 expression [20,21]. Additionally, the retrospective design of the study may introduce bias in data collection and patient selection. These limitations highlight the need for further research to validate the findings and address potential biases [22-24].

Future studies should incorporate survival analysis to better understand the prognostic significance of p53 overexpression in CRC [25,26]. Longitudinal studies tracking patients through treatment and post-treatment periods could provide deeper insights into how p53 expression influences treatment responses, particularly in patients undergoing targeted therapies or immunotherapy. Further investigation into the relationship between p53 expression, MSI status, and molecular subtypes, such as KRAS and BRAF mutations, is also warranted. This would help clarify how these markers interact and influence treatment strategies, potentially leading to more personalized approaches for CRC patients.

## Conclusions

This study provides valuable insights into the clinicopathological characteristics of CRC and the role of p53 expression as a potential biomarker for tumor progression. Elevated p53 expression was significantly associated with advanced tumor stages, larger tumor size, and lymph node involvement, highlighting its role in promoting CRC aggressiveness. The presence of molecular mutations, such as BRAF and KRAS, correlated with p53 overexpression, suggesting a complex interplay in CRC pathogenesis. The findings underscore the importance of IHC analysis of p53 in evaluating CRC prognosis and guiding therapeutic strategies, warranting further investigation into its prognostic implications.
